# Encapsulated Cellular Implants for Recombinant Protein Delivery and Therapeutic Modulation of the Immune System

**DOI:** 10.3390/ijms160510578

**Published:** 2015-05-08

**Authors:** Aurélien Lathuilière, Nicolas Mach, Bernard L. Schneider

**Affiliations:** 1 Division of Neurology, Department of clinical Neuroscience, Geneva University Hospitals, 1205 Geneva, Switzerland; E-Mail: aurelien.lathuiliere@hcuge.ch; 2 Neurodegenerative Studies Laboratory, Brain Mind Institute, Ecole Polytechnique Fédérale de Lausanne (EPFL), 1015 Lausanne, Switzerland; 3 Department of Oncology, Geneva University Hospital and Medical School, 1205 Geneva, Switzerland; E-Mail: nicolas.mach@hcuge.ch

**Keywords:** cellular implants, genetic engineering, encapsulation, passive immunization, recombinant antibodies, Alzheimer’s disease, cytokine, adjuvant, cancer vaccine

## Abstract

*Ex vivo* gene therapy using retrievable encapsulated cellular implants is an effective strategy for the local and/or chronic delivery of therapeutic proteins. In particular, it is considered an innovative approach to modulate the activity of the immune system. Two recently proposed therapeutic schemes using genetically engineered encapsulated cells are discussed here: the chronic administration of monoclonal antibodies for passive immunization against neurodegenerative diseases and the local delivery of a cytokine as an adjuvant for anti-cancer vaccines.

## 1. Encapsulated Cell Technology for Therapeutic Protein Delivery

### 1.1. General Concept of Cell Encapsulation

The implantation of genetically engineered cells to continuously deliver biomolecules including recombinant proteins into a host organism is an attractive concept in terms of *ex vivo* gene therapy for the treatment of chronic diseases. Although the transplantation of autologous cells can be considered for some applications, the use of a single, well-characterized source of cells applicable to a large number of recipients is a key advantage for standardization of the procedure. Indeed, after genetic engineering, it is often crucial to assess the amount and the quality of the therapeutic molecule produced by the cells, which requires a careful characterization of the cell source prior to implantation. Therefore, techniques have been developed to transplant renewable cells in allogeneic or even xenogeneic conditions.

**Figure 1:**
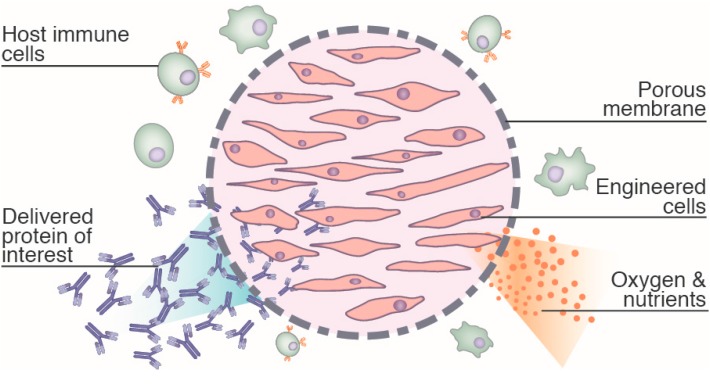
Overall concept of encapsulated cell technology.

In the absence of immunosuppressive drugs, the transplantation of allogeneic tissues or cells induces an immune response in the host, rapidly leading to transplant failure and rejection. To overcome immune rejection, the use of the encapsulated cell technology (ECT) has been proposed [1]. ECT is based on the confinement of the implanted cells within a polymeric permeable membrane (Figure 1). Hence, the cells to be implanted are loaded in a device, which can be inserted into the host tissue and retrieved through a simple surgical procedure. The selective porosity of the membrane allows for the diffusion of nutrients and oxygen to support the survival of the cells inside the device. Therapeutic proteins and metabolic by-products of the implanted cells are also released outside from the device by passive diffusion. Importantly, the polymer membrane provides a mechanical barrier that prevents any direct contact with the host immune cells. With the immunoprotection provided by this barrier, cells can be implanted in allogeneic conditions. The long-term survival of allogeneic cells using ECT has indeed been demonstrated in various implantation sites [2,3,4]. Additionally, cell encapsulation even allows for the successful engraftment of xenogeneic cells, provided the cells are implanted in immunoprivileged sites [5,6].

ECT has been investigated for the transplantation of therapeutic cells naturally secreting bioactive products, such as hormones and trophic factors, or cells genetically engineered for *de novo* expression of a protein of interest [7,8,9,10]. Most of the research efforts in ECT have been aimed at developing artificial organs to recover endocrine pancreatic function, with the long-term objective of treating diabetic patients. However, this technology is also well adapted for the chronic delivery of therapeutic biological products into sites that are not suitable for repeated injections, which has opened a broad spectrum of potential applications in the central nervous system (CNS) and the eye. For instance, this approach has been clinically tested for the intrathecal delivery of neurotrophic factors in patients affected by amyotrophic lateral sclerosis [5], or for the intraocular administration of trophic factors against macular degeneration [11].

Two different approaches have been developed to encapsulate cells. With “microencapsulation” technology, cells are enclosed in capsules with a sub-millimeter size, each containing a cluster of a few thousand cells. The “macroencapsulation” format is based on the utilization of large biocompatible polymer devices, with a size typically in the centimeter range, and which contain several million cells. Macroencapsulation devices can be retrieved to halt the treatment and allow for better control over the dose of biomolecules administered. Therefore, we will focus on macroencapsulation, which is the most advanced format for the applications discussed in the present review.

### 1.2. Macroencapsulation Systems

The main advantage of using a macrocapsule is the option to retrieve the device through a simple surgical procedure. Therefore, the treatment can be halted in the case unwanted side effects occur. The option to retrieve the implanted cells confined within the device addresses major safety concerns, such as the risk of uncontrolled proliferation of grafted cells inside the host tissue. Nonetheless, such medical devices must fulfill important criteria to comply with clinical standards, including the use of medical grade biomaterials and adequate sterilization methods. Other aspects detailed below are critical for the development of macroencapsulation devices to support the survival of implanted cells over the long term.

**Figure 2:**
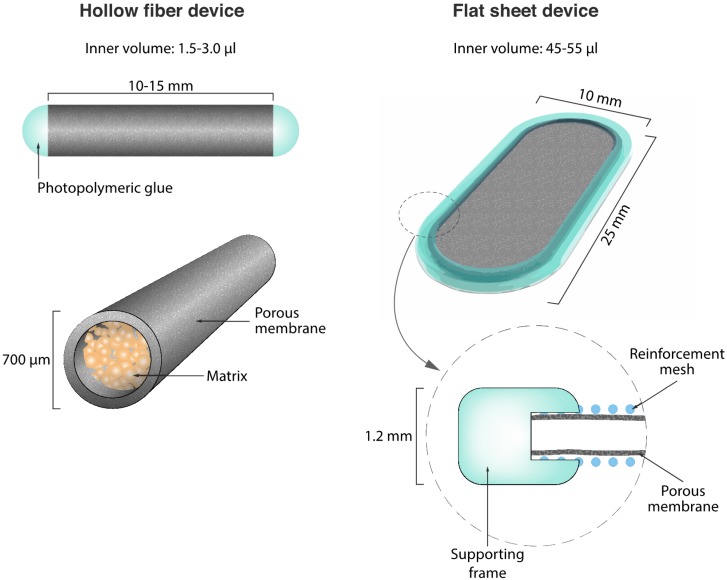
Main types of devices for macroencapsulation.

To adapt several million cells, macrocapsules require rational design optimization. For instance, a spherical geometry cannot be considered for individual capsules, as it fails to maximize the passive diffusion of factors essential for cell survival, such as oxygen and nutrients. Instead, the main geometries are either cylindrical hollow fibers or planar diffusion chambers (Figure 2). Hollow fiber devices usually consist of a manufactured porous membrane, which can be sealed using photo-polymerizing medical glue once the cells are loaded inside the fiber lumen. The device is typically linked to a tether to facilitate explantation. Hollow fiber devices are well adapted to implantation into the eye [12], the subcutaneous tissue [13] and the CNS, either intrathecally [5] or intraparenchymally [14]. Hollow fibers have also been directly connected to the host bloodstream by establishing an arterio-venous anastomosis. In this system, the grafted cells are confined in an external chamber lining the permeable membrane, and are therefore maintained near the bloodstream to facilitate molecular exchanges [15]. However, intravascular devices were associated with a high risk of hemorrhages and thromboembolic events, which necessitates chronic administration of anticoagulants. Therefore, the FDA has stopped their development [16].

Recently, an alternative “flat sheet” geometry has been reported [17]. This device is based on a polymer frame, which is used to assemble two sheets of porous polymer membranes, and two sheets of mesh placed on the external face of the permeable membrane. The role of the mesh is to mechanically reinforce the device and favor neovascularization in close proximity to the permeable membrane. The flat sheet geometry is characterized by a large inner chamber volume, which has the advantage of providing higher cell capacity than hollow fibers. Flat sheet devices are typically used for subcutaneous or intraperitoneal implantation [17,18].

The porous membrane is the most important component of the encapsulating device. The main role of the membrane is to provide a mechanical barrier that prevents direct cell-to-cell contact with host immune cells, while allowing passive diffusion of soluble factors essential to grafted cell survival. Most of the membranes used in macroencapsulation are made of thermoplastic polymers, such as poly(ether-sulfone), the copolymer of polyacrylonitrile and polyvinyl chloride (PAN–PVC), polyurethane, polysulfone, polypropylene, polyvinylidene difluoride (PVDF), polytetrafluoroethylene (PTFE) or expanded PTFE (ePTFE) [19]. Microporous membranes have pore sizes in the 0.1–1.0 µm range, and therefore allow all macromolecules to passively diffuse. In contrast, semi-permeable membranes have pore sizes that are small enough to let only certain soluble molecules pass through. Membrane porosity is characterized by the molecular weight cutoff (MWCO). The MWCO, which is expressed in Daltons, is defined by the molecular weight of a soluble molecule, for which the membrane can reduce the diffusion by 90% in standard conditions; the lower the MWCO, the smaller the membrane pore size. However, as the size of the pores typically follows a Gaussian distribution, the MWCO is only approximately defined for most of the polymer membranes. Hence, the passive diffusion of soluble molecules within a range of molecular weight values higher than the nominal MWCO cannot be excluded. Moreover, this diffusion is also affected by other molecular factors including charge, hydrophobicity or steric hindrance [16].

On principle, the degree of immunoprotection might depend on membrane permeability to large macromolecules mediating humoral immunity, such as antibodies or complement proteins. The necessity of limiting the diffusion of these factors has been highly debated [20]. Overall, microporous membranes that mainly prevent cell-to-cell contacts have been successfully used in allogeneic conditions [2,17,21,22,23]. However, the expression of exogenous proteins by the implanted cells [24], or massive necrotic cell death inside the device can dramatically affect the long-term survival of encapsulated cells [25]. Membrane perm-selectivity may be more critical to immunoprotection in xenogeneic conditions, which typically requires immunosuppression to avoid rejection of the encapsulated cells within a few days [26]. Furthermore, membrane permeability has been shown to affect the amount of therapeutic product released [27]; opting for a large pore size membrane can therefore maximize therapeutic protein delivery. The diffusion of oxygen from the blood vessels lining the device towards the cells inside the device is another key factor for the survival of encapsulated cells at high density. Therefore, membrane thickness and porosity should be selected to maximize oxygen supply.

For long-term implantation, the membrane, as well as other device components, must have sufficient mechanical resistance to withstand shear stress and prevent crushing. For instance, hollow fiber membranes are prone to bending and kinking, which may cause device failure. In order to provide mechanical reinforcement, different strategies have been developed, such as adding a titanium coil in the hollow fiber [28], or an external thermoplastic reinforcement mesh [17,29].

Devices are usually seeded with adherent cells that progressively grow in the capsule to form a dense cell mass. Maximal cell density primarily depends on the diffusion of oxygen and nutrients inside the device. Typically, when dense cell clusters are formed, oxygen and nutrients do not efficiently diffuse to the center of the cluster, leading to a necrotic core. Intracellular factors released from the necrotic cell core can be toxic to neighboring cells, and may also diffuse into the surrounding host tissue, where they elicit a detrimental immune response. To facilitate the three-dimensional expansion of adherent cells in the device and control cell density [25], different types of supporting matrices have been included in cell encapsulation systems. A wide range of scaffolds have been tested, including “bio-inspired” matrices classically composed of laminin or collagen [30], polymeric hydrogels [17] or biologically inert material such as glass, plastic beads and polyvinyl alcohol (PVA) foam [25,31,32].

### 1.3. Genetic Engineering of Cells

For therapeutic protein delivery by ECT, genetically engineered cells have to survive and maintain stable transgene expression over periods of several days to months, depending upon the envisioned application. As metabolic conditions inside the device are restrictive, only a few cell types are compatible with ECT. Several factors must be considered when selecting optimal cell types for ECT. Encapsulated cells must be able to stably produce the recombinant protein at therapeutic levels. For many potential applications, reaching the therapeutic level is a limiting factor for this technology. It is therefore critical to identify adequate cell types that (i) are amenable to genetic engineering; (ii) can produce functional proteins with proper posttranslational modifications; and (iii) are capable of sustained protein secretion even in restrictive metabolic conditions. Moreover, the cell source should be phenotypically stable and easy to expand in regular culture conditions. The possibility of multiplying a clonal population from a single cell is a clear advantage for clinical application, which requires the full characterization of the implanted cells. For long-term implantation, cell sources with strong contact inhibition are preferred, as cells will slow down or halt proliferation once the device is filled. For allogeneic implantation in humans, it is essential to use renewable cells of human origin, as ECT will allow for the use a single cell source applicable to all patients.

Different strategies have been used to genetically engineer cell lines dedicated to ECT. While regular transfection techniques are very efficient in most of the cell sources considered for ECT, the use of viral vector technologies provides an effective alternative for cell types that are less permissive to transfection, or for expression systems that necessitate better control over the number of inserted copies. Lentiviral vectors are widely used for their ability to efficiently integrate into both proliferative and post-mitotic cells. They preferentially integrate the transgene into transcriptionally active regions of the host cell genome, enhancing the stability of transgene expression. In addition, their use for *ex vivo* gene therapy has been validated in clinical trials. In particular, genetic engineering of implantable cell lines with lentiviral vectors provides several advantages over regular transfection for the expression of complex proteins such as recombinant antibodies. A dual lentiviral vector strategy has recently been developed to generate ECT-compatible myoblast cell lines secreting recombinant monoclonal antibodies [33]. Further details on this system are described in the Section 2.1.2 on passive immunization.

The most recent developments in ECT suggest that this technology could be a promising alternative for the delivery of complex recombinant proteins to modulate immune responses. Despite the fact that ECT has been shown to effectively deliver recombinant proteins in humans [5,6,14], clinical application has been limited for various reasons. Often, promising results in preclinical rodent models could not be successfully translated to the clinic because the amount of protein delivered by ECT could not be scaled up to humans. For other applications, the efficacy of delivered factors was uncertain. Although neurotrophic factors could be chronically delivered to the central nervous system by ECT, it remains unclear whether they would be able to provide effective neuroprotection against the targeted disorders, which included amyotrophic lateral sclerosis, Parkinson’s and Huntington’s diseases. Lately, novel applications have emerged, for which ECT is used to modulate the activity of the immune system via the acute or chronic delivery of key effector proteins. These applications will be reviewed in the following section.

## 2. Encapsulated Cell Technology as a Strategy to Modulate the Immune System

Immunotherapy refers to the modulation of the natural immune response in order to treat or prevent diseases. The two main therapeutic strategies are active and passive immunization. Active immunization involves the delivery of an antigen that can be associated with an adjuvant in order to elicit an immune response in the host organism. Passive immunization refers to the direct administration of antibodies specifically targeting an antigen.

ECT has key features that can be used to modulate the immune system. Chronic delivery of recombinant proteins provides a useful alternative to bolus injection of recombinant antibodies for passive immunization. Alternatively, encapsulated cells can locally deliver therapeutic immunoactive factors such as anti-inflammatory interleukins, as proposed for the treatment of rheumatoid arthritis [34], or cytokine adjuvants for active immunization. Here, we will review two such applications that are based on ECT: (1) passive immunization against neurodegenerative diseases by chronically delivering specific antibodies targeting misfolded proteins and (2) active immunization against cancer cells by locally releasing an adjuvant cytokine.

### 2.1. Passive Immunization against Chronic Neurodegenerative Disorders

#### 2.1.1. Therapeutic Antibodies for Passive Immunization

The therapeutic potential of exogenous antibodies was first proposed by a group of scientists including Paul Ehrlich, Emil von Behring (Nobel prize laureates in 1908 and 1901, respectively), Erich Wernicke and Shibasaburo Kitasato at the Institute for Infectious Disease in Berlin in the late 19th century. They found that animals inoculated with fully virulent forms of diphtheria or tetanus could be cured when administered the serum of animals exposed to attenuate forms of the infectious agent. Their investigations eventually led to the first immunotherapies based on the injection of animal serum. Antiserum therapies dramatically reduced the mortality caused by infectious diseases during the early 20th century. The therapeutic use of antibodies essentially relied on serum derived from immunized animals, until recombinant protein technology emerged. Nowadays, highly specific antibodies can be engineered to precisely interfere with various disease pathways.

Therapeutic antibodies are probably the most promising class of therapeutics in the pharma industry. In 2013, four of the five best-selling pharmaceutical products were biologics [35], and among biologics, monoclonal antibodies (mAbs) is the leading class of therapeutics. In 2011, mAbs sales increased by 10% in the US to reach $20.3 billion [36], which accounts for one third of total sales of biologics. This dramatic expansion is mainly due to major technological advancements in antibody engineering, improving safety and efficacy profiles. With the increasing number of ongoing clinical trials and market approvals, novel mAbs will likely elicit more therapeutic breakthroughs [37]. However, some key questions and technical challenges remain to be solved for future mAb therapies.

Most recombinant protein therapeutics are produced using mammalian cell cultures. During the past twenty years, cell culture capacity has dramatically increased to meet the demand for recombinant proteins, including mAbs. By the beginning of 2015, 42 mAbs had been approved by regulatory agencies [38]. Six of them were approved in 2014 and six more are currently undergoing regulatory review with action expected from the FDA or EMA in 2015 [39]. Currently, there are 39 phase II/III or phase III clinical trials using therapeutic antibodies [39]. This illustrates the great enthusiasm for passive immunization therapies, which were first developed as treatment against cancer and auto-immune disorders, and more recently found applications in the treatment of other conditions, such as osteoporosis (denosumab) [40], or high blood cholesterol (evolocumab and alirocumab) [41,42,43]. Despite the recent developments in biomanufacturing, which have improved the yield of recombinant protein production, the supply may become an issue if numerous new biological drug candidates are approved in the coming years.

As compared to other classes of drugs, mAbs offer the advantage of a selective mode of action, with lower risk of side effects. However, the bioavailability of these large proteins with both hydrophilic and hydrophobic moieties remains limited in some tissues and body compartments, such as the CNS. Hence, in order to reach therapeutic levels, mAbs are typically administered through repeated intravenous injections at high doses (in the 0.1–1.0 g/dose range). High dosing further increases the cost burden of mAb therapies, which are already expensive because of the complex manufacturing and quality controls [44]. The median US wholesale price for mAbs reaches $8000 per gram [44], and the typical cost for a continuous treatment is near $35,000 per year (e.g., natalizumab for the treatment of multiple sclerosis) [45].

From a pharmacological point of view, the repeated administration of mAbs at high doses also has some limitations. Antibodies can elicit neutralizing immune responses, which may affect their efficacy [46,47]. Although the humanization of mAbs has significantly reduced their immunogenicity [48], chemical alterations such as oxidation, deamination, polymerization or fragmentation can dramatically increase their propensity to induce immune responses [49,50]. At high concentrations, the exposure of hydrophobic regions, or particular charge distribution over the protein, may cause antibody denaturation and aggregation, which is a well-described physical factor enhancing immunogenicity [51,52]. Furthermore, post-translational modifications (PTM) including glycosylation, affect both the immunogenicity and stability of mAbs. PTM also modulate antibody effector function, which is linked to affinity for binding partners (reviewed in [53]). Culture conditions, protein purification process or storage conditions are known to induce variability in the PTM of mAbs [54,55,56].

Finally, the expansion of medical applications for therapies based on biopharmaceuticals brings some ethical questions. The use of mAb therapies for chronic and highly prevalent diseases may impose huge economic burdens on healthcare systems. In particular, mAb therapies are tested for the treatment of neurodegenerative diseases where they may interfere with pathological events associated with the misfolding of proteins such as amyloid β (Aβ), Tau, α-synuclein or SOD1 (reviewed in [57,58]). If successful, mAb therapies could be proposed for chronic, highly prevalent neurodegenerative conditions such as Alzheimer’s disease (AD) [59], Parkinson’s disease [60], tauopathies [61] or amyotrophic lateral sclerosis [62]. However, the use of mAbs for the treatment of these pathologies may be limited by the cost to the healthcare system.

Although mAb-based therapies can be very efficient, several issues should be addressed to make them fully accessible to a large portion of the population. The development of innovative delivery technologies, such as ECT, may represent a way to overcome some of these limitations and provide novel therapeutic options. In particular, the use of cellular implants could be an alternative when mAbs need to be administered either chronically or to poorly accessible tissues.

#### 2.1.2. Passive Immunization Using Encapsulated Cell Transplants for the Prevention of Alzheimer’s Disease

Alzheimer’s disease is the most prevalent neurodegenerative disorder. It is responsible for 50% to 75% of all dementia cases. Because its incidences increases with aging, from less than 10% of people over 60 to more than 30% of people over 80 years old, the number of patients affected is expected to dramatically increase in aging societies [63]. In 2050, AD is anticipated to affect 115 million people worldwide [64]. AD pathophysiology is mainly characterized by the deposition of Aβ aggregates and neurofibrillary tangles composed of hyperphosphorylated tau. Robust genetic evidence supports the “amyloid cascade hypothesis”, which postulates that Aβ accumulation is a primary pathological event subsequently leading to neuronal dysfunction, tau hyperphosphorylation and neuronal death [65]. It is now evident that Aβ accumulation starts years, even decades, before the first clinical symptoms of AD become apparent [66,67]. As a consequence, interfering with early events of the Aβ cascade has become the focus of the research efforts exploring possible therapies against AD. In this context, passive immunization to reduce Aβ accumulation is the most advanced disease-modifying strategy towards clinical development. The administration of mAbs targeting Aβ efficiently clears brain amyloid pathology in various transgenic AD mouse models [68,69,70,71], through mechanisms that are not fully elucidated yet (reviewed in [72]). These findings have prompted clinical testing in AD patients, and the results of two phase-III clinical trials using different anti-Aβ mAbs have been released. Mild to moderate AD patients intravenously treated for 18-months with bapineuzumab, an antibody binding specifically to the *N*-terminus of Aβ, did not show any effect on cognitive function or functional outcome measurements [73]. Nevertheless, the analysis of biomarkers demonstrated target engagement, as bapineuzumab reduced the level of phospho-tau (a marker of neurodegeneration) in the cerebrospinal fluid, and decreased the rate of amyloid deposition measured by Pib-PET amyloid imaging [73,74,75]. Solanezumab is a humanized monoclonal IgG1, which is directed against the central part of Aβ [76]. In a phase-III trial, solanezumab did not slow down cognition decline in mild to moderate AD patients treated for 80 weeks. Nevertheless, a significant benefit to cognition was observed in a subgroup of mild AD patients [77]. It is therefore plausible that anti-Aβ immunotherapy should be applied at an early stage of the disease to impact on disease progression before irreversible neuronal damage has occurred [78]. Three secondary prevention trials were later initiated to test this possibility: the Alzheimer’s Prevention Initiative (API) [79], the Anti-Amyloid Treatment in Asymptomatic AD (A4) and the Dominant Inherited Alzheimer’s Network trial (DIAN).

In this context, the use of ECT has been proposed for the delivery of anti-Aβ antibodies to prevent AD in patients with signs of amyloid accumulation. In a proof-of-concept study, hollow fiber macroencapsulation devices were seeded with myoblasts genetically engineered to secrete a single-chain variable antibody fragment (scFv) antibody fragment directed against the *N*-terminus of the Aβ peptide. Following intracerebral implantation, the scFv antibody chronically produced by ECT efficiently reduced Aβ production and deposition in a transgenic mouse model of AD [4]. Moreover, this treatment had a beneficial effect on behavioral deficits associated with the amyloid pathology. However, the implantation of an encapsulation device directly into the brain parenchyma is an invasive procedure, which represents a major obstacle to clinical development in presymptomatic AD patients.

**Figure 3:**
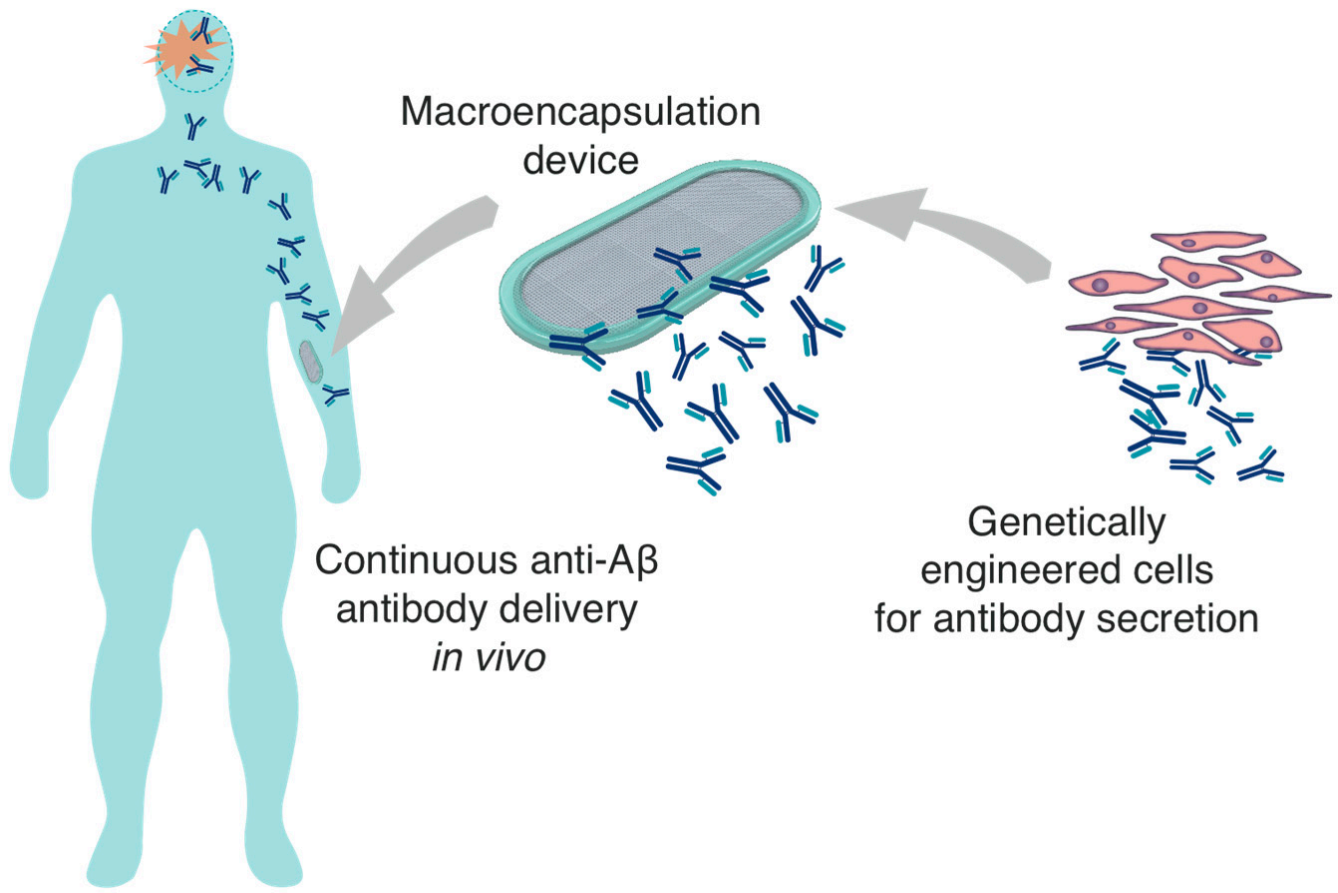
*In vivo* delivery of encapsulated cells genetically engineered to produce anti-Aβ antibodies following implantation in the subcutaneous tissue.

An alternative strategy with a less invasive implantation procedure would be better adapted to disease prevention in patients at risk of AD. A possible approach is to implant the encapsulated cellular implant in the subcutaneous tissue, for the systemic delivery of the therapeutic mAb, with the aim of inducing the clearance of the amyloid pathology in the brain (Figure 3). To establish such an approach, several major technical challenges have to be overcome: (i) it is critical to generate stable cell lines suitable for ECT and that secrete significant amounts of anti-Aβ antibodies; (ii) a large capacity cell encapsulation device has to be developed that can support the long-term survival of the implanted cells in the subcutaneous tissue; (iii) it is important to demonstrate that the implanted ECT device can secrete anti-Aβ antibodies for several months, reaching therapeutic levels of recombinant antibody measurable in the plasma, to ultimately achieve beneficial effects on brain amyloid pathology. Our recent work has focused on the rational development and optimization of methods to demonstrate the feasibility and efficacy of this approach.

A dual lentiviral vector strategy was used to generate myoblast cell lines secreting a monoclonal antibody against Aβ [33]. Using separate lentiviral constructs encoding either the light or heavy chains of the mouse IgG2a protein, populations of mouse myoblasts were obtained that stably produced high levels of recombinant IgG with a proper glycosylation pattern. By exposing cells to incremental doses of the two vectors, populations of transduced C_2_C_12_ myoblasts were shown to secrete up to 8 pg/cell/day of recombinant IgG. Individual clonal cell lines were isolated from these stably transduced myoblast populations, with antibody production levels >10 pg/cell/day. The highest secretion reached 29 pg/cell/day, which represents a nearly 40-fold increase in cell-specific productivity compared to classical transfection methods using a similar expression cassette. Importantly, these myoblast cell lines maintained their ability to survive long-term following implantation in ECT devices.

In order to increase the number of transplanted cells and thereby maximize the amount of antibody delivered, a novel flat sheet device has recently been reported for subcutaneous implantation [17]. This encapsulation device is assembled through a reproducible and highly controlled ultrasonic welding system. The ECT device has a smooth shape avoiding shear stress in the surrounding tissue. The inner chamber can contain up to several millions myoblast cells, which can be loaded in the device via a dedicated port. In order to promote the three-dimensional expansion of cells initially seeded at low density, the myoblasts are loaded within a biodegradable polymeric polyethylene glycol hydrogel matrix. After careful optimization of the initial cell seeding conditions, this tissue engineering approach was shown to support the survival of genetically engineered cells at high density for up to one year, a time frame compatible with the chronic administration of recombinant antibodies.

In mice, a preliminary experiment demonstrated that an anti-Aβ IgG antibody produced by ECT could be chronically detected in the plasma of the subcutaneously implanted animals at levels reaching 50 μg/mL for up to 19 weeks *in vivo* [33]. Ongoing work suggests that similar levels of mAb circulating in the plasma can be sustained for more than 10 months after device implantation. These observations validate ECT technology combined with myogenic cells for the long-term delivery of anti-Aβ mAbs. Now, it is important to determine if the antibodies produced by ECT can enter the CNS and reduce the amyloid pathology. Overall, ECT can be adapted to produce recombinant mAbs *in vivo*, and could be used for a number of applications, as an alternative to repeated bolus injections.

### 2.2. Active Immunization against Cancer Cells

Historically, the implementation of vaccination or active immunization is the medical development with the most beneficial impact on human health. Vaccination is typically achieved by inoculating inactive forms of the pathogenic microorganism, or immunogenic parts thereof, with the objective of evoking an immune response and preventing or limiting its pathogenic effects. Most vaccines against infectious agents elicit an antibody response to preventively block primary infection. Recent developments in immunology and vaccinology are expanding the repertoire of applications for the treatment of preexisting disease, by developing vaccines against pathogens leading to persistent infections, such as *Mycobacterium tuberculosis*, hepatitis C virus and human immunodeficiency virus [80,81,82]. Active immunization against self-derived antigens can be used in a vast spectrum of non-infectious disorders including neurodegenerative disorders such as Alzheimer’s, Parkinson’s or prion diseases [83,84,85], auto-immune disorders and atherosclerosis [86]. Another key therapeutic target is cancer, which is the focus of a novel application for ECT.

Vaccines against non-infectious disorders typically target abnormal autologous proteins, which are often associated with disease. Indeed, tumor cells display specific antigenic proteins that are differently expressed compared to normal cells. These proteins can be either mutated, overexpressed or reactivated by the pathological condition, or carry abnormal PTM [87]. Most tumor-associated antigens are derived from self proteins (altered self) and are therefore likely to be less immunogenic, or even tolerogenic [88]. To overcome this tolerance and induce an immune response, considerable research efforts have been made to identify powerful adjuvants.

#### 2.2.1. Cytokines as Adjuvants for Immunization against Tumor Cells

The generation of an effective immune response relies on both innate and acquired immunity. The initial stimulation of innate immunity is considered critical. The major antigen-presenting cells (APC) are dendritic cells and macrophages, which reside in peripheral tissues and process antigens. APC can uptake soluble antigens (via pinocytosis), but also antigens bound to pattern recognition receptors (PRR) [89]. A multiplicity of PRR are expressed at the surface of APC, where they recognize pathogen-associated molecular patterns (PAMP), often referred as “danger signals” [90]. The binding of antigens to PRR activates the APC, inducing the expression of the major histocompatibility complex (MHC) for antigen presentation, co-stimulatory molecules including CD40, CD80 and CD86, as well as cytokines and chemokine receptors (e.g., CCR7) [91]. The type of danger signal will determine the co-stimulatory factors expressed by APC [92,93], which govern the resulting immune response [94]. Anti-infectious vaccines and adjuvants typically induce differentiation of naïve T-cells into T helper type 2 (Th2) cells, which express IL-4, IL-5 and IL-13, and promote humoral immunity [95]. In anti-tumor vaccination, it is preferred to induce Th1 T cells, which express IFN-γ, IL-2 and TNF-α, and mediate cellular immunity, including cytotoxic T lymphocytes that target and lyse malignant cells [96].

A better mechanistic understanding of innate immunity, especially of the implicated cytokines, has driven the discovery and optimization of immune adjuvants. Direct control over local cytokine levels has been proposed to modulate and enhance anti-tumor immunity. Candidate cytokines include IL-2, IFN-α, -β, -γ, TNF-α and granulocyte-macrophage stimulating-factor (GM-CSF), probably the most studied and promising cytokine adjuvant.

#### 2.2.2. Anti-Cancer Active Immunization Using Encapsulated Cells Secreting Granulocyte-Macrophage Stimulating-Factor (GM-CSF)

GM-CSF is a secreted 23 kDa highly glycosylated single-chain protein (128 amino acids) that was first isolated from mouse lung conditioned culture medium for its ability to induce the proliferation of bone marrow cells forming colonies of both granulocytes and macrophages [97]. It is produced by a variety of cell types including macrophages, T-cells, mast cells, fibroblasts, and endothelial cells [98,99]. GM-CSF is expressed in response to pro-inflammatory signals including TNF-α, IL-6 or lipopolysaccharides [100,101], and induces the proliferation of various cells, such as pluripotent progenitors, macrophage progenitors, granulocytes and megakaryocytes, as well as the erythroid lineage [102]. Moreover, GM-CSF stimulates the maturation of APC from their precursors, and thereby directly modulates the immune response to antigens [103].

Immunization with irradiated tumor cells genetically modified to express GM-CSF leads to the recruitment of APC to the site of immunization. GM-CSF induces APC to express MHC-II and T-cell co-stimulatory molecules such as B7-2 or CD40 [104,105], thereby enhancing antigen presentation and T-cell activation [106]. Vaccination with GM-CSF was more effective than other cytokines in inducing a powerful, long-lasting anti-tumor immunity in several cancer mouse models [107].

The injection of tumor cells modified to secrete GM-CSF has rapidly been transferred to the clinic. Clinical trials were performed in patients with various types of cancer, such as renal cell carcinoma [108], melanoma [109,110], prostate [111], lung [112] and pancreas cancer [113]. These clinical studies demonstrated the ability of GM-CSF to induce an effective T-cell and/or antibody anti-tumor immune response when secreted locally at the site of cancer cell injection. The use of autologous tumor cells as a vehicle for GM-CSF administration has the advantage of exposing the patient in the same time to the repertoire of tumor-specific antigens. However, autologous cell lines need to be established from tumors collected by surgery prior to genetic engineering, which might result in highly variable and poorly predictable rates of GM-CSF secretion. These practical constraints are likely to limit the clinical development of this cellular vaccination technique.

An alternative approach is to genetically engineer generic allogeneic tumor cell lines for GM-CSF expression. This would indeed facilitate the production, storage and modification (*i.e.*, irradiation) of vaccines [114]. However, there is a great molecular heterogeneity among tumors of different origins [115,116,117,118], which suggests that similar cancer-inducing mutations may generate cell subclones that later diverge and develop phenotypic diversity [119]. Hence, the repertoire of antigens is likely to be different between generic tumor cell lines and the patient’s tumor, which may decrease the efficacy of the vaccine and allow for the development of secondary, immune-resistant tumors. In addition, allogeneic cells will be rapidly eliminated by the host immune system, which may limit the production of GM-CSF at the site of injection and therefore decrease the adjuvant effect. These vaccines have nevertheless been evaluated in clinical trials for different types of cancer, with mitigated results when used in monotherapy (reviewed in [120]). Another alternative is to replace gene transfer by local co-injection of recombinant GM-CSF protein (rGM-CSF), together with autologous tumor cells or peptides. However, cancer vaccines based on the co-injection of rGM-CSF have shown inconsistent results. Some studies even reported a deleterious effect [121,122]. It has been proposed that fine-tuning the administered dose of rGM-CSF is key to achieving vaccine efficacy (reviewed in [123]). Systemic exposure to large quantities of rGM-CSF can be detrimental, leading to the recruitment of tolerogenic immune cells such as myeloid suppressor cells. In a murine cell-based immunization model, additional systemic administration of rGM-CSF abolished the long-lasting, specific, protective anti-tumor immune response obtained by locally releasing GM-CSF using genetically engineered cells [124]. Furthermore, a comparative study has demonstrated that the local production of GM-CSF is more effective than bolus subcutaneous injection of the recombinant cytokine in mounting an effective immune response [125]. Therefore, local exposure to GM-CSF for a few days may be needed to recruit and activate APC [126].

**Figure 4:**
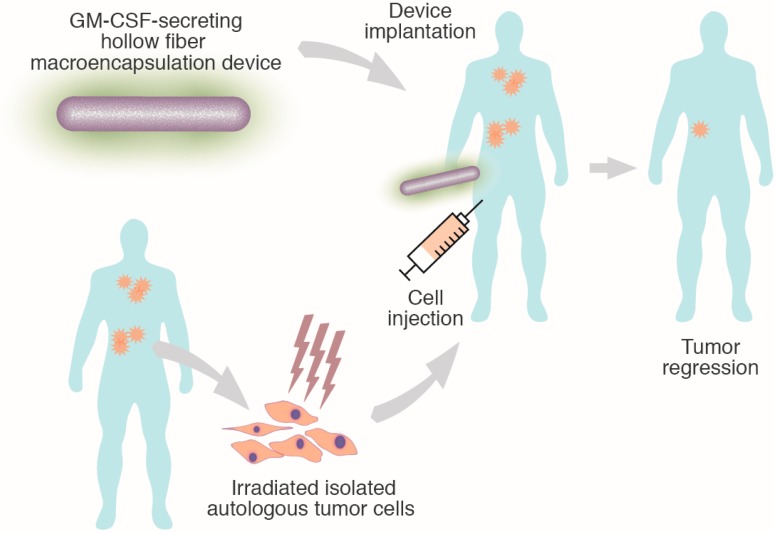
Therapeutic autologous tumor cells vaccination using granulocyte-macrophage stimulating-factor (GM-CSF)-expressing encapsulated cells as a strong adjuvant.

The use of ECT was recently proposed for the local delivery of GM-CSF combined with tumor cell vaccination [13]. Here, the overall concept is to use an ECT device for the implantation of a “bystander” allogeneic cell line engineered for the production of GM-CSF. The GM-CSF-secreting device is implanted in the subcutaneous tissue, in order to produce the cytokine in the same site as tumor cell injection, with the goal of generating a local adjuvant effect. In a proof-of-concept study, GM-CSF secreting cell lines were generated and were shown to survive within a hollow fiber encapsulation device implanted in the subcutaneous tissue [13]. Encapsulated GM-CSF-expressing cells were able to deliver significant amounts of GM-CSF *in vitro* and *in vivo*, and induced strong inflammatory reactions in the surrounding tissue. Hence, ECT may overcome some major limitations of vaccination via direct injection of autologous or allogeneic GM-CSF-secreting tumor cells. As encapsulated cells are used as an adjuvant system to locally deliver GM-CSF, there is no need to genetically modify the patient-derived tumor cells. Therefore, autologous cells can be used for vaccination, providing the full repertoire of tumor antigens. In addition, the use of the encapsulation device containing known amounts of GM-CSF-secreting cells provides some control on the delivered dose of cytokine, and protects the cells from immediate rejection. For these two reasons, the combination of these two technologies may enhance the efficacy of the anti-tumor vaccine. This strategy is currently being tested in a first-in-man phase-I clinical trial (NCT02193503, www.clinicaltrials.gov). The trial is enrolling patients suffering from advanced metastatic solid tumors. The vaccination protocol combines the subcutaneous implantation of MVX-ONCO-1, an ECT device containing a human cell line secreting GM-CSF, with the injection of autologous irradiated cells isolated from the patient’s tumor (Figure 4).

## 3. Perspectives

ECT is an advanced biotechnological approach for the continuous administration of therapeutic recombinant proteins, either locally or systemically. It has already been well accepted for clinical investigation, offering promise for novel treatments against prevalent, chronic diseases. This concept has great potential, in particular for applications based on molecules that can profoundly modulate the activity of the immune system. ECT is indeed an effective technique for the chronic delivery of recombinant antibodies, either systemic or targeted to poorly accessible tissues, with the objective of specifically interfering with pathogenic molecular processes. With the constant expansion of the arsenal of therapeutic recombinant antibodies, this versatile delivery technology will open novel opportunities for therapeutic applications. In addition, the use of ECT has great potential to overcome the limitations associated with the injection of recombinant cytokines to locally produce an adjuvant effect, providing a novel technology in the challenging field of anti-cancer immunization. These innovative immunomodulatory treatments based on ECT will be evaluated in both preclinical and clinical settings in the coming years.
